# Eukaryotic Cell Capture by Amplified Magnetic *in situ* Hybridization Using Yeast as a Model

**DOI:** 10.3389/fmicb.2021.759478

**Published:** 2021-11-01

**Authors:** Fabiola Bastian, Delphine Melayah, Mylène Hugoni, Nora M. Dempsey, Pascal Simonet, Marie Frenea-Robin, Laurence Fraissinet-Tachet

**Affiliations:** ^1^DTAMB, Université Claude Bernard Lyon 1, Bât. Gregor Mendel, Villeurbanne Cedex, France; ^2^Université Lyon, Université Claude Bernard Lyon 1, CNRS, INRAE, VetAgro Sup, UMR Ecologie Microbienne, Villeurbanne, France; ^3^Institut Universitaire de France (IUF), Paris, France; ^4^Université Grenoble Alpes, CNRS, Grenoble INP, Institut Néel, Grenoble, France; ^5^Université Lyon, Université Claude Bernard Lyon 1, Ecole Centrale de Lyon, INSA Lyon, CNRS, Ampère, UMR 5005, Ecully, France

**Keywords:** eukaryotic cells, magnetic nanoparticles, *Saccharomyces cerevisiae*, magnetic *in situ* hybridization, HCR, cell fishing

## Abstract

A non-destructive approach based on magnetic *in situ* hybridization (MISH) and hybridization chain reaction (HCR) for the specific capture of eukaryotic cells has been developed. As a prerequisite, a HCR-MISH procedure initially used for tracking bacterial cells was here adapted for the first time to target eukaryotic cells using a universal eukaryotic probe, Euk-516R. Following labeling with superparamagnetic nanoparticles, cells from the model eukaryotic microorganism *Saccharomyces cerevisiae* were hybridized and isolated on a micro-magnet array. In addition, the eukaryotic cells were successfully targeted in an artificial mixture comprising bacterial cells, thus providing evidence that HCR-MISH is a promising technology to use for specific microeukaryote capture in complex microbial communities allowing their further morphological characterization. This new study opens great opportunities in ecological sciences, thus allowing the detection of specific cells in more complex cellular mixtures in the near future.

## Introduction

In recent years, research in microbial ecology has truly taken off. This spectacular breakthrough is mainly due to rapid technological advances such as meta-omics, which have significantly increased our ability to study microbial communities from complex environments and their function in various ecosystems (Morris et al., 2002; Hiraoka et al., 2016; Grumaz et al., 2020; Sehnal et al., 2021). An ecosystem is a huge reservoir of yet uncharacterized biodiversity especially concerning microeucaryotes, which play a key role in ecology, for example in bacterial predation or recalcitrant organic matter degradation. Although detection of eukaryotic microorganisms in natural ecosystems using high-throughput sequencing is well documented (e.g., Barbi et al., 2014; Sutcliffe et al., 2019; Mundra et al., 2021), deciphering the microbial biodiversity in ecosystems and understanding the underlying complexity of a community’s structure and function remain important challenges.

As a long-standing technique, Fluorescent *In Situ* Hybridization (FISH) has been and still is widely used to visualize complete intact cells (for a clinical review see Berg et al., 2016; Frickmann et al., 2017). Several modifications have allowed the FISH procedure to be applied to different models and have inspired the development of many other techniques since the 1980s (Bauman et al., 1980; Amann et al., 1990; Wallner et al., 1993; Wang et al., 2019). For instance, microsystems and fluorescence-based monitoring through powerful platforms can be used to separate and observe entire cell staining with simple diagnostic fluorescent dyes, especially when investigating the heterogeneity of cellular systems (Pivetal et al., 2014a). As a recently developed method, Fluorescent *in situ* DNA-hybridization chain reaction (HCR-FISH) may additionally offer the opportunity to overcome the main problem of FISH, i.e., low intensity of the signal, due to low rRNA content found in some environmental microorganisms (Yamaguchi et al., 2015; Jia et al., 2021). However, cell isolation by FISH or HCR-FISH requires a coupling with flow cytometry, which can be used for some but not all environments. For example, unicellular microorganisms cannot be directly isolated from soils or sediments due to the presence of many mineral and calcareous impurities present in these environments.

Magnetophoresis-based separation is increasingly used as an alternative to fluorescent activated cell sorting (FACS) in various biomedical (Yu et al., 2018) and environmental applications (Zhu et al., 2020). Magnetic sorting devices are less sophisticated, more compact, and less expensive than FACS. Moreover, high gradient magnetic separation platforms or integrated micromagnets (Osman et al., 2013) have proven very effective in capturing cells with minimal labeling (i.e., cells labeled with nanoparticles carrying weak magnetic moments) (Plouffe et al., 2015).

Recently, the magnetic procedure HCR-MISH (MISH = Magnetic *in situ* hybridization) has been proposed as a sensitive method for the isolation by direct magnetic capture of whole intact bacterial cells from complex environments, using a combination of *in situ* hybridization and HCR amplification (Royet et al., 2018). The principle of HCR-MISH is to use a magnetic field to capture specific cells onto which superparamagnetic nanoparticles are attached by a nucleic acid probe (either DNA or RNA), the length of which is enlarged and amplified inside and outside the cell by HCR. In addition, micro-magnet arrays integrated in microfluidic channels are powerful tools to selectively extract magnetically labeled cells (Osman et al., 2013). While the MISH technique has been till now successfully applied to the isolation of specific labeled bacterial cells (Stoffels et al., 1999; Pivetal et al., 2014a,b; Royet et al., 2018), enlarging it to the isolation of eukaryotic cells will offer a real opportunity to describe the microeukaryotic diversity.

In MISH, long probes are used; obtained either from 23S RNA fragment synthesis (Stoffels et al., 1999; Pivetal et al., 2014a) or from a long artificial HCR-amplified DNA fragment. Due to the limited permeability of the cell wall, only part of the probe is linked to its intracellular target site (Zwirglmaier et al., 2003), while the remaining part is located outside the cell, which allows anchorage of magnetic nanoparticles on accessible biotinylated sites.

Here we describe a procedure that allows grafting of super-paramagnetic nanoparticles onto targeted micro-eukaryotic cells using yeast (*Saccharomyces cerevisiae*) as a model, exploiting magnetism for their subsequent isolation using a micro-magnet array. By applying HCR-MISH on an artificial mixture comprising prokaryotic and eukaryotic cells and by using a universal 18S eukaryotic probe, we selectively isolated the eukaryotic fraction, thus delivering a promising method usable to target eukaryotic microbial communities.

## Materials and Methods

### Strains and Culture

*Saccharomyces cerevisiae* BY4741 strain (MATa, his3Δ1, leu2Δ0, met15Δ0, ura3Δ0) (Euroscarf) and *Escherichia coli* DH5α strain [F^–^ *endA1 glnV44 thi-1 recA1 relA1 gyrA96 deoR nupG purB20* φ80d*lacZ*ΔM15 Δ(*lacZYA-argF*)U169, hsdR17(*r*_*K*_^–^*m*_*K*_^+^), λ^–^] (Promega) were used as eukaryotic and bacterial cells, respectively, in the HCR-MISH experiments. Yeast cells were cultivated in YPG (yeast extract 10 gL^–1^, peptone 20 gL^–1^, and glucose 20 gL^–1^) at 30°C. Bacterial cells were grown in low salt Luria–Bertani Broth (Duchefa Biochemie) at 36°C. All microbial cells were cultivated with a 150 rpm-orbital shaker, thus providing active growing cells at the logarithmic growth phase.

### Probe *in silico* Analysis

The universal eukaryotic probe used in this study was Euk516 antisense i.e., Euk516R 5′- ACCAGACTTGCCCTCC -3′ (Diez et al., 2001) targeting eukaryotic cells. This choice was based on previous work conducted on soils (Lehembre et al., 2013). The specificity of the probe was tested *in silico* using the Silva SSU r138 database (4th December 2020, Pruesse et al., 2007).

### Hybridization Chain Reaction-Magnetic *in situ* Hybridization Principle

**FIGURE 1:**
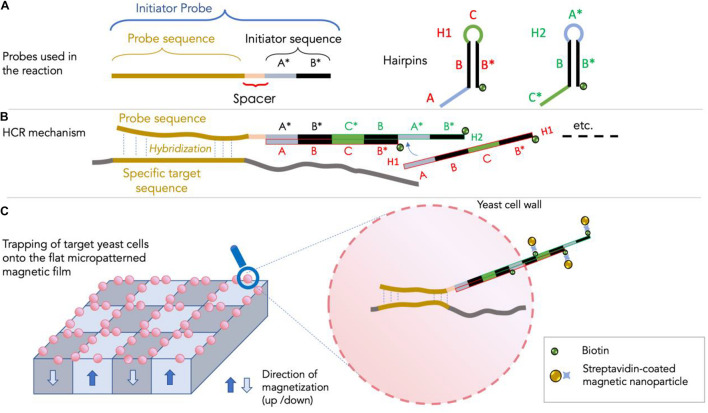
The HCR-MISH approach (adapted from Royet et al., 2018). In panel **(A)**: the three probes (one specific initiator and two DNA hairpin H1 and H2 probes) used in the HCR-MISH approach are shown. Note that the specific initiator probe contains a specific sequence for the specific MISH hybridization, along with a spacer and an initiator sequence for the HCR amplification. The hairpin H1 and H2 probes are composed of three short sequences: **(A–C)**. In panel **(B)**: the HCR amplification step showing the overlapping H1 and H2 chain hybridization. In panel **(C)**: the magnetic labeling of the yeast cells as the ultimate result of the newly synthetized double-strand DNA composed of H1 and H2 overlapping probes, that are located outside the cell.

The principle of the method involves the use of three DNA probes (Figure 1): an initiator probe and two DNA hairpin probes, referred to as H1 and H2 (Dirks and Pierce, 2004). The initiator probe is composed of 5′–3′ of four sequences: (i) a 16 bp-long antisense sequence specific to the target 18S rRNA sequence, (ii) a short (5 bp-long) spacer sequence, and (iii) a sequence containing two (13 bp-long) A and B sequences which allow triggering the opening of the DNA hairpin of the H1 probe and subsequent self-assembly of the two amplifier probes H1 and H2 during HCR (Figure 1B) as shown in Royet et al. (2018) and modified for this study. The self-assembling of H1 and H2 sequences during HCR allows the creation of a long DNA fragment potentially crossing the cell, reaching a size of several thousand base pairs inside and outside the cell. As shown in Figure 1C, the H1 and H2 amplifier probes are biotinylated for subsequent attachment outside the cell of streptavidin-coated superparamagnetic nanoparticles. The different probe sequences are presented in Table 1. For this first proof of concept, we used as specific sequence the antisense of the universal eukaryotic primer Euk516R, targeting the 18S rRNA and rDNA of eukaryotes including yeasts (Diez et al., 2001). The main steps of the protocol behind the use of HCR-MISH on whole eukaryotic cells consists in: (i) performing a cell fixation, to keep cell morphology, followed by an enzymatic treatment for partial yeast cell wall hydrolysis to allow probes to enter into the cell, (ii) hybridizing target genomic DNA and/or target RNA transcripts using the initiator probe, and (iii) carrying out a chain reaction of hybridization events introducing H1 and H2 probe amplifiers.

**TABLE 1:** Sequences and probes used in the experiment.

**Names**	**Sequences (5′–>3′)**
Euk516-MISH (with spacer)	CCGAATACAAAGCATCAACGACTAGAAAAAACCAGACTTGCCCTCC
H1 probe*	TCTAGTCGTTGATGCTTTGTATTCGGCGACAGATAACCGAATACAAAGCATC
H2 probe*	CCGAATACAAAGCATCAACGACTAGAGATGCTTTGTATTCGGTTATCTGTCG

*^∗^: the H1 and H2 probes were 5′ labeled with biotin.*

### Hybridization Chain Reaction-Magnetic *in situ* Hybridization Hybridization

Active growing cells were harvested by centrifugation at 4000 *g* for 1 min and washed in sterile 1× PBS buffer (130 mM NaCl, 7 mM Na_2_HPO_4_, 3 mM NaH_2_PO_4_, pH 7.2). Then, either yeasts, bacterial cells or an appropriate ratio of eukaryotic/bacterial cells were fixed in 3% (w/v) extemporaneously prepared paraformaldehyde in 1× PBS solution for 1 h at 30°C, and then pelleted and washed at room temperature in 1× PBS buffer. Cell samples were then incubated in hybridization buffer (20 mM Tris–HCl, 0.9 M NaCl, 0.01% SDS, 50% (v/v) formamide) for 30 min at 30°C, washed and suspended in 1× PBS at room temperature. A partial yeast cell-wall hydrolysis was carried out by adding 10U zymolyase enzyme (Zymo Research), incubating for 15 min at 30°C and washing cells twice in 1× PBS buffer. Then cells were suspended in 100 μL hybridization buffer containing the initiator probe at 0.5 μM final concentration. Hybridization was performed at 37°C for at least 3 h. Cells were then washed twice in pre-warmed (55°C) 1× PBS buffer and suspended in 100 μL amplification buffer consisting of 50 mM Na_2_HPO_4_, 0.9 M NaCl and 0.01% (v/v) SDS. Prior to amplification, each H1 and H2 probe was denatured separately for 90 s at 95°C and then cooled for 30 min at room temperature. Next, the amplifying mix containing both the denatured biotinylated H1 and H2 probes was prepared as follows: H1 and H2 amplifier probes were mixed and added to the cell samples (for a final 2.5 μM concentration in the amplification buffer). HCR amplification lasted 2 h at 46°C. Afterward, samples were washed twice with ice-cold 1× PBS. Finally, 10 μL commercial streptavidin-coated superparamagnetic beads (Miltenyi Biotech, Streptavidin MicroBeads, diameter 50 nm, concentration not provided by the manufacturer) were added. After an overnight incubation at 4°C, cells were washed and suspended in 1× PBS. The HCR-MISH protocol is summarized in the Supplementary Table 1.

### Staining and Microscopy

Cell suspension (100 μL) was stained by adding 0.2 μL of 0.1 mg.ml^–1^ ethidium bromide (EthBr) and incubating for 5 min at room temperature. Cells were washed in 1× PBS, harvested by centrifugation and re-suspended in 1× PBS. After 5 min, stained cells (10 μL) were deposited onto a micro-magnet array, integrated or not in a micro-fluidic device (see next section), and observed using a Zeiss Axio Imager equipped with a DsRed filter. Images were acquired using a Zeiss AxioCamMR3 camera and Axiovision software.

### Micro-Magnet Array

A hard magnetic film of NdFeB was deposited on a Si wafer and patterned using thermo-magnetic patterning, as previously described (Dumas-Bouchiat et al., 2010). The resulting structure consists of a chessboard pattern of alternatively magnetized square domains of size 100 × 100 μm^2^. The magnetic field (>1 T) and field gradient (>10^5^ T/m) produced in the vicinity of this micro-magnet array are exploited to trap magnetically labeled cells on its surface, organizing them in a square pattern corresponding to the regions of maximum stray field. The microfluidic integration of such micro-magnet arrays was developed following the technique described by Osman et al. (2013). Briefly, a 50 μm thick dry photoresist layer (LAMINAR^®^ E92200 dry film photopolymer) was laminated by hand onto a glass substrate before exposure to ultra violet light through a photomask bearing the microchannel geometry (using KLOE UV-KUB exposure and masking system, wavelength 365 nm). The exposed negative photoresist film was then developed in a Na_2_CO_3_ solution at a concentration of 0.85% (w/w), heated to 35°C. PDMS preparation consisted in mixing Sylgard 184 silicone base and curing agent (purchased from Neyco) at 10:1 mass ratio. After vacuum degassing, the mixture was poured over the PDMS master and allowed to cure in an oven at 80°C for 2 h. After peeling off the PDMS replica, two holes were punched at each end of the microchannel.

#### Microchannel Bonding

The same PDMS mixture as described above was diluted with Heptane (Sigma-Aldrich) to obtain a 4% (w/w) PDMS solution. The dilute solution was spin-coated onto the magnet surface at 4500 rpm for 1 min (using a Spin 150, SPS-Europe) and baked at 80°C for a few hours to enable solvent evaporation and PDMS curing. The PDMS microchannel and the PDMS-coated substrate were then sealed together after exposing both surfaces to air plasma treatment (Expanded Plasma Cleaner, Harrick Plasma).

#### Flow Control Setup

A NE-4000 Multi-Phaser Double Syringe pump was used to control the flow rates. For this purpose, syringe needles were connected to PTFE tubing (1/32″ ID × 1/16″ OD) directly inserted into the PDMS port holes of 1.25 mm diameter.

## Results

### Probe *in silico* Analysis

The sequence Euk516R (Diez et al., 2001) used in this study for targeting *S. cerevisiae* 18S rRNA genes, following the MISH procedure, is a non-degenerated 16 bp-long eukaryotic universal sequence and corresponds with 100% homology to the antisense of the *S. cerevisiae* 18S rRNA gene sequence. This sequence has already been used as one of the eukaryotic universal primer pairs in several eukaryotic microorganism diversity studies conducted in different environments, especially in soils (Bailly et al., 2007; Damon et al., 2012; Lehembre et al., 2013). *In silico* analysis of the Silva SSU r138 database (4th of December 2020) confirmed that the Euk516R sequence is very specific to the *Eukaryota* domain as it is able to target 82.6% of eukaryotic 18S rRNA gene sequences, only 2% of archaeal sequences and 0% of bacterial ones. Indeed, this sequence is localized in a region very well conserved among eukaryotic 18S rRNA genes and is able to target a significant number of eukaryotes, including unicellular microeucaryotes. The main eukaryotic phyla which can be targeted with this sequence according to our analysis are presented in the Supplementary Table 2. This probe covers 93.1% of Fungi present in the Silva database and is 100% identical to the *S. cerevisiae* 18S rRNA gene. Moreover, as shown in this table, this sequence is also very well conserved among unicellular microeukaryotes belonging to the SAR super-phylum (i.e., 89.7% of *Alveolata*, 90.8% of *Rhizaria*, and 94.6% of *Stramenopiles*) whereas the *Excavata* phylum is much less represented (1.3%) and *Amoebozoa* about half represented (67.4%).

### Hybridization Chain Reaction-Magnetic *in situ* Hybridization on Eukaryotic Cells

**FIGURE 2:**
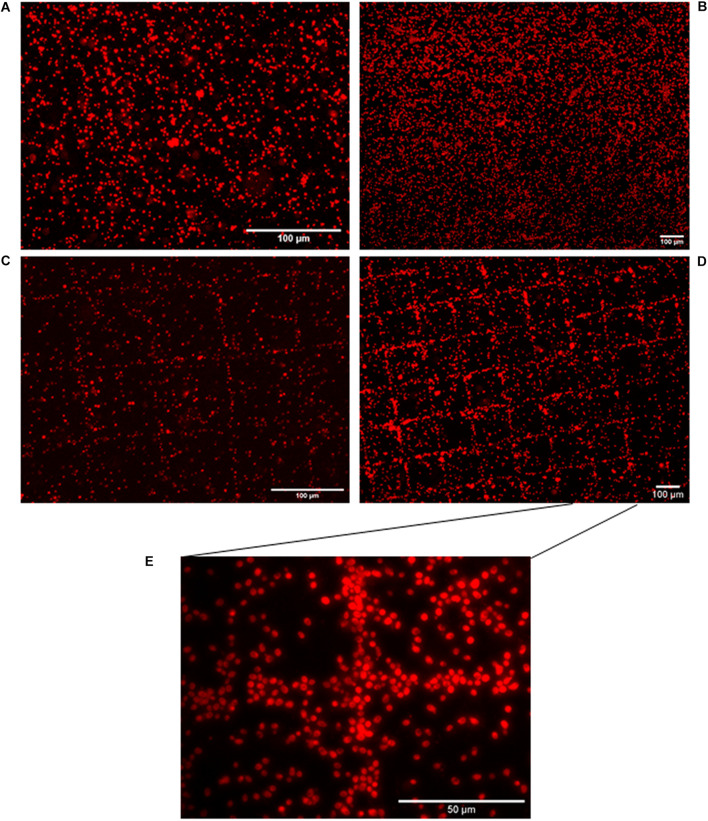
Eukaryotic cell distribution patterns following HCR-MISH. Yeast cells from calibrated samples were subjected to complete treatment apart from incubation with superparamagnetic nanoparticles (10× magnification) **(A)**. Yeast cells were in contact with just biotinylated probes (H1 and H2), in the absence of the initiator sequence (including the 18S probe) and then were placed on the micro-magnet array (10× magnification) **(B)**. H1 probe was solely used, i.e., without H2, images show a few square patterns (20× magnification) **(C)**. Complete treatment of HCR-MISH using both amplifiers and the specific probe 18S, the patterns obtained are far more distinct 10× **(D)**. Same treatment as **(D)**, but at higher magnification, yeast cells can be individually distinguished: 50× **(E)**.

Limitation of *in situ* hybridization efficiency due to the structure of the cell wall is well known, as exemplified for bacteria (Moter and Göbel, 2000). Yeast cell permeability assays using enzymatic treatment with zymolyase prior to hybridization monitoring by FISH allowed us to address this issue. We consequently adapted our HCR-MISH protocol to include this pretreatment step to loosen cell wall integrity prior to hybridization. Several control experiments were then performed to test the feasibility of HCR-MISH on eukaryotic cells using yeast as a model. Firstly, yeast cells were subjected to the complete technique apart from incubation with superparamagnetic nanoparticles (Figure 2A). No reaction indicating any attraction of hybridized yeast onto the micro-magnet array and their subsequent assembly into square patterns could be observed. This confirms that the treated yeast cells are not attracted by the magnetic field, as can naturally happen under specific conditions for certain microeucaryotes (Kim et al., 2020). Secondly, the random distribution of yeast on the micro-magnet array obtained after treatment with superparamagnetic nanoparticles and biotinylated probes (H1 and H2) but without specific initiator probe (Figure 2B) allowed to verify that neither the superparamagnetic nanoparticles nor the H1 and H2 biotinylated probes bind specifically to the yeast cells. This result also indicated that the nanoparticles did not get internalized by the fixed yeast cells. The following step of this experiment was to test the complete technique with only the H1 hairpin probe, i.e., no H2 hairpin probe, in order to determine whether HCR is necessary. In this case, the images showed a few square patterns with very thin cell strips (Figure 2C), suggesting that some nanoparticles could have been grafted after H1 hybridization. This result showed that: (i) the specific initiator probe is functional, and (ii) H1 hairpin probe could be sufficient for the labeling of a few cells, but the efficiency is low. However, the patterns obtained with the complete technique, including HCR amplification, allowed to detect clear regular square patterns with thick cell strips where multiple yeast cells were captured (Figures 2D,E for higher magnification and Supplementary Video). The labeling efficiency can be estimated by counting the yeast cells trapped on the square patterns. The percentage of capture was above 82.8%. It was estimated by counting yeast cells on the whole surface of three different micro-magnet arrays, onto which three different samples were deposited.

This last experiment revealed the feasibility of the HCR-MISH technique and that HCR amplification is essential for high efficiency of the technique, as it greatly improves the yeast cell capture yield. However, while above 82.8% of yeast cells were trapped on the magnetic chessboard, a small percentage remained un-trapped and randomly dispersed on the surface, probably because they were unlabeled or too weakly labeled. As shown in the video (Supplementary Video), cells can be trapped under continuous flow inside the microfluidic device, meaning that labeled cells can be separated from unlabeled ones: When the sample is injected in the device, only the target (magnetically functionalized) cells are trapped, while the rest of the mixture moves toward the device outlet. Then, as previously studied by our team (Osman et al., 2013; Pivetal et al., 2014a), the trapped cells can be recovered by simply increasing the flow rate. These results demonstrate the feasibility of the HCR-MISH method to capture eukaryotic cells such as yeast.

### Specificity of Eukaryotic Hybridization Chain Reaction-Magnetic *in situ* Hybridization Capture

**FIGURE 3:**
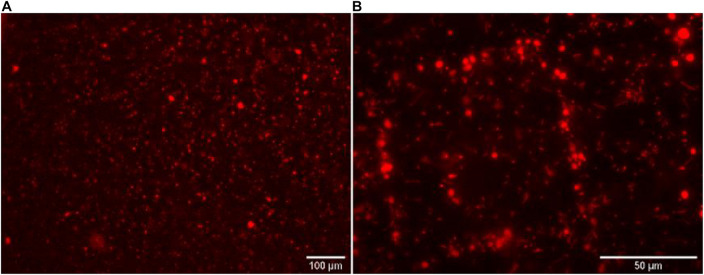
The eukaryotic HCR-MISH specificity as evaluated for target eukaryotic cells in an artificial mixture. *E. coli* cells were the control test without the specific 18S probe under 50× magnification **(A)** and with the 18S probe **(B)** using a concentration of bacteria 30 times higher than the yeast.

The next step of our work aimed at testing the HCR-MISH specificity against other organisms, such as bacteria. This was investigated with an artificial cell mixture comprising the yeast *S. cerevisiae*, as the eukaryotic model cell, and the bacteria *Escherichia coli*, as the prokaryotic model organism, in different proportions (1:10, 1:30, and 1:100, respectively). All mixtures follow the same treatment. In a control experiment without the 18S rRNA gene specific initiator probe (Figure 3A), a random distribution of yeast and bacteria on the micro-magnet array was observed. On the other hand, after complete treatment, specific yeast cell attraction was visible on the micro-magnet array: the larger yeast cells followed the square patterns while the much smaller bacteria were randomly distributed. This was observed whatever the bacterial concentration used, 10 times higher than yeast or 30 times higher (as shown in Figure 3B). This demonstrated that in this experiment, cell attraction by HCR-MISH capture from a prokaryotic and eukaryotic cell mixture is yeast specific, even though bacteria were introduced in excess.

## Discussion

The aim of this work was to provide a simple and cost-effective approach that can be used for trapping and fishing whole morphologically intact eukaryotic cells using magnetic nanoparticles with a specific universal eukaryotic probe. We demonstrated that this procedure can be used with microfluidic platforms. We focused on a combination of HCR-MISH with magnetic cell sorting using high performance micro-magnets integrated into microfluidic devices. In this study, we used *S. cerevisiae* eukaryotic cells as a model.

In eukaryotes, DNA probes coupled with magnetic nanoparticles have been largely employed to investigate different RNA with specific hybridization, with good results and great applications, but to our knowledge, this approach was dedicated only to lysed eukaryotic cells and not to complete cells, as in the present study (Koo et al., 2016; Li et al., 2016; Pang et al., 2016; Zhang et al., 2016; Yuan et al., 2017). In a medical context, the combination of magnetic nano-probes and HCR (HCR-MISH) has been reported for the electrochemical determination of multiple eukaryotic micro RNAs simultaneously in cell lysates (Yuan et al., 2017) or to capture RNA biomarkers from mutated cells in cancer diagnosis (Pang et al., 2016), but as far as we know not to capture whole intact eukaryotic cells. In a microbial ecological context, the development of this technique for eukaryotic microorganisms fills the gap left by other molecular biology techniques and all the techniques of -omics for isolating cells from yet unknown (and mostly uncultured), eukaryotic microorganisms. These can be detectable through orphan environmental 18S sequences which cannot be robustly affiliated, or through environmental cDNAs isolated by screening for a functional phenotype but with no hit in data bases and thus not affiliated at all (Lehembre et al., 2013; Ziller et al., 2016).

Cells labeled by the method described here are morphologically intact but not viable due to the fixation step performed with paraformaldehyde, which aims at denaturing and achieving crosslinking of proteins. Nevertheless, the integrity of fixed cells is preserved and they remain genetically exploitable for subsequent morphological characterization and different genomic applications (Bussolati et al., 2011), such as trapping whole cells, to detect whole parasites in animals or humans (Calderon et al., 2015).

Cell or tissue isolation has been a first prerequisite to characterize cell function or genome specificity or to gain a deeper insight into cellular particularities or heterogeneities within populations, which are important requirements for an ecological understanding of microbial processes and for many other biological applications (Brehm-Stecher and Johnson, 2004). For instance, in a global health context, understanding antibiotic resistance in eukaryotic cells (Watamoto et al., 2011; Sandai et al., 2016; Ahmad Khan et al., 2020) or pathogen detection (Loll-Krippleber et al., 2015; Haridas et al., 2017; Albadr et al., 2018) are common examples where the perception of cellular heterogeneities is needed. So far, flow-cytometry and microscopy imaging (with its limits) has been the most popular method to study cells individually (Pivetal et al., 2014b; Leonard et al., 2016) or from a specific taxonomic or functional community in both medical and environmental contexts. As an example of the potential application of HDR-MISH to eukaryotes, one could consider studying thermite gut microbiota comprising protists, most of which are unique. Although this symbiosis has long been intriguing to researchers of both basic and applied sciences, its detailed mechanism remains unclear due to the enormous complexity and the non-cultivability of its microbiota (Hongoh, 2011; Carpenter et al., 2013). In plants with economic significance, one possible application could be to isolate *Plasmodiophora brassicae* (belonging to Cercozoa), the causal agent of many canola clubroot diseases, which cannot be cultured outside of its host (Holtz et al., 2018). The use of fluorescent cell sorters is tempered by the problem of auto-fluorescence, which does not occur with magnetic sorters. With MISH, prior isolation or enrichment of the targeted cells in pure culture is not required, which broadens its application to uncultured eukaryotic microorganisms. The MISH method allows single intact cell isolation directly from environments and is thus highly appropriate to further characterize trapped cells, morphologically by microscopy or genetically by whole genome sequencing of single cells or a few cells. The feasibility of this experiment opens new prospects in cell tracking in various ecosystems such as dental, lung or aquatic ones. Associating whole cell trapping with single-cell sequencing technologies could provide a powerful tool for assessing relevant information in extremely rare but precious cells. Combining all the “-omics” and single cell resolution, will bring to the forefront an unexplored landscape and may address questions that remain unanswered in diverse fields of biological and ecological sciences (Alam et al., 2018). Consequently, alternative methods such as MISH remain useful to directly observe and characterize yet unknown microorganisms, some of them supporting part of the functional biodiversity.

In our work we used the 18S rRNA probe, which is a generalist probe available to analyze the whole cells belonging to a specific clade in environments. Other probe functions or clade-specificity could be used to trap microeukaryotes belonging to a functional community. Future research should focus on the development and application of this technique on other eukaryotic cells and cell fishing from complex samples from different environments.

## Conclusion

The present study reports a new method combining hybridization chain reaction and magnetic *in situ* hybridization for tracking and separating eukaryotic cells using commercial superparamagnetic nanoparticles. We show that yeast can be selectively trapped from an artificial mix of microorganisms. We have demonstrated static trapping and flow-based separation of eukaryotic-labeled cells. Since this approach was previously validated on bacteria by Royet et al. (2018), these new results have enlarged the toolbox available for microbiologists to study complex environmental samples.

This method will need further studies to adapt to each type and specificity of eukaryotic cells, but it provides a new tool to track cells without needing to lyse them, allowing the characterization of the whole cell by morphological analysis or whole genome single-cell sequencing. The combination of HCR and magnetic *in situ* hybridization shows great promise for environmental research, as it appears to be applicable to both bacteria (Royet et al., 2018) and eukaryotic cells (this present work).

## Data Availability Statement

The original contributions presented in the study are included in the article/Supplementary Material, further inquiries can be directed to the corresponding author.

## Author Contributions

FB acquired, analyzed, or interpreted the data for the work, and drafted the manuscript. ND provided the micro magnet arrays. MH analyzed or interpreted the data for the work and revised it critically for important intellectual content. DM and PS revised it critically for important intellectual content. MF-R and LF-T made substantial contributions to the conception or design of the work and revised it critically for important intellectual content. All authors contributed to the article and approved the submitted version.

## Conflict of Interest

The authors declare that the research was conducted in the absence of any commercial or financial relationships that could be construed as a potential conflict of interest.

## Publisher’s Note

All claims expressed in this article are solely those of the authors and do not necessarily represent those of their affiliated organizations, or those of the publisher, the editors and the reviewers. Any product that may be evaluated in this article, or claim that may be made by its manufacturer, is not guaranteed or endorsed by the publisher.
