# Supplementary material for: Eukaryotic Cell Capture by Amplified Magnetic in situ Hybridization Using Yeast as a Model
Source: Front Microbiol. 2021 Nov 1;12:759478. doi: 10.3389/fmicb.2021.759478 (PMC8591292; doi:10.3389/fmicb.2021.759478)
Supplement: Supplementary file 1 [file Table_1.docx]

**SUPPLEMENTARY DATA**

**Supplementary table S1.** Synthetic eukaryotic HCR-MISH protocol.

Step 1. Cell fixation

1-1. Fix cells with fresh paraformaldehyde 3% PBS solution at 30°C for 1 h.

1-2. Wash cells in PBS buffer (130 mM NaCl, 7 mM Na_2_HPO_4_, 3 mM NaH_2_PO_4_, pH 7.2) at room temperature.

1-3 Pellet cells by centrifugation at 10,000 rpm.

Step 2. Cell wall and plasma membrane permeabilization

2-1. Prepare hybridization buffer [20 mM Tris-HCl, 0.9 M NaCl, 0.01% SDS, X% formamide].

2-2. Wash cells in PBS buffer.

2-3. Treat the samples with 100µL hybridization buffer, incubate at 30°C for 30 minutes.

2-4. Wash in PBS buffer and recover in 1 mL PBS buffer.

2-5. Treat the sample with zymolyase (5 U/µL) 15 min at 30°C.

2-6. Wash in PBS buffer.

Step 3. Hybridization of the specific initiator probe

3-1. Recover the sample in 100µL hybridization buffer.

3-2. Add the probe at a final concentration of 0.5 μM.

3-3. Incubate for at least 3 h at 37°C.

3-4. Wash with pre-warmed PBS buffer at 55°C.

Step 4. Preparation of amplifier probes [to be done during the incubation step (3-3)]

4-1. Prepare amplification buffer [50 mM Na_2_HPO_4_, 0.9 M NaCl and 0.01% SDS].

4-2. Prepare each amplifier probe (H1 and H2) in the amplification buffer (5 μM each) in an independent tube, heat 90 sec at 95°C and cool 30 min at room temperature.

Step 5. *In situ* DNA-hybridization chain reaction (HCR)

5-1. Add the two amplifier probes at a final concentration of 2.5 μM for each probe.

5-2. Incubate 2 h at 46°C.

5-3. Wash in cold PBS buffer, 4°C (to prevent probe dissociation).

Step 6. Magnetic nanoparticle binding

6-1. Mix 10 µL of nanoparticles (50 nm streptavidin coated superparamagnetic beads, Miltenyi Biotec).

6-2. Incubate overnight at 4°C.

6-3. Wash in PBS buffer, and re-suspend in an appropriate amount of PBS buffer.
