# Supplementary material for: Eukaryotic Cell Capture by Amplified Magnetic in situ Hybridization Using Yeast as a Model
Source: Front Microbiol. 2021 Nov 1;12:759478. doi: 10.3389/fmicb.2021.759478 (PMC8591292; doi:10.3389/fmicb.2021.759478)
Supplement: Supplementary file 2 [file Table_2.docx]

**SUPPLEMENTARY DATA**

**Supplementary table S2.** *In silico* analysis of the specificity of 18S rRNA gene probe Euk516R used for MISH hybridization targeting eukaryotic rDNA sequences. This analysis was carried out against the Silva SSU r138 database (4^th^ of December 2020) with the *Eukaryota* domain divided in twelve different taxonomic phyla, some of which are presented in this table with some ecologically relevant sub-divisions including in particular micro-eukaryotes.

| **Taxonomic levels** | **Coverage (%)** | **Specificity (%)** |
| --- | --- | --- |
| ***Eukaryota* domain** | 82.6 | 99.9 |
| ***Amorphea*** | 8.8 | 95.3 |
| *Amoebozoa* | 67.4 | 90.0 |
| *Obazoa* | 84.3 | 95.2 |
| *Opisthokonta* | 84.3 | 95.2 |
| *Holozoa* | 80.5 | 93.3 |
| *Nucletmycea* | 93.1 | 91.6 |
| *Aphelidea* | 79.6 | 89.9 |
| Fungi | 93.1 | 91.6 |
| *Nucleariidae* and *Fonticula* group | 100 | 89.9 |
| ***Archaeplastida*** | 74.5 | 90.9 |
| ***Cryptophyceae*** | 20.8 | 89.9 |
| ***Discoba*** | 28.3 | 89.9 |
| ***Excavata*** | 1.3 | 89.9 |
| ***Haptophyta*** | 97.3 | 89.9 |
| ***Incertae Sedis*** | 9.6 | 89.9 |
| *Ancyromonadida* | 90.0 | 89.9 |
| *Apusomonadidae* | 100 | 89.9 |
| *Mantamonas* | 100 | 89.9 |
| *Palpitomonas* | 100 | 89.9 |
| *Palustrimonas* | 100 | 89.9 |
| *Telonema* | 94.7 | 89.9 |
| **NAMAKO-1** (uncultured eukaryotes) | 100 | 89.9 |
| **Picozoa** | 94.1 | 89.9 |
| **SA1-3C06** (uncultured eukaryotes) | 0 | 0 |
| **SAR** | 91.0 | 92.5 |
| *Alveolata* | 89.7 | 91.5 |
| *Rhizaria* | 90.8 | 90.3 |
| Stramenopiles | 94.6 | 90.5 |
| **Uncultured** | 100 | 89.9 |
